# Supplementary figures and images for: Early detection of COVID-19 outbreaks using human mobility data
Source: PLoS One. 2021 Jul 20;16(7):e0253865. doi: 10.1371/journal.pone.0253865 (PMC8291683; doi:10.1371/journal.pone.0253865)

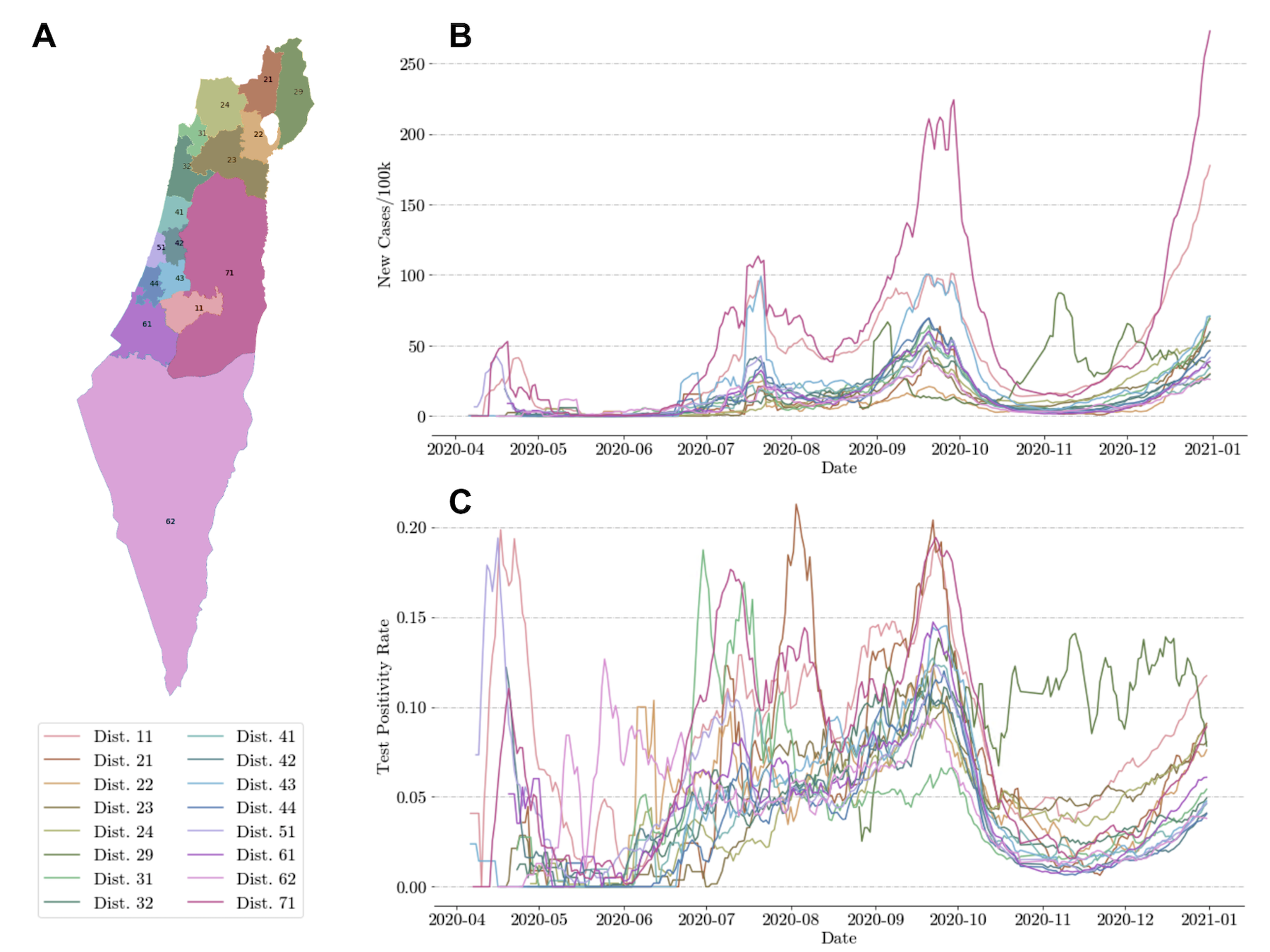

Supplement: S1 Fig — (TIF) [file pone.0253865.s001.tif]

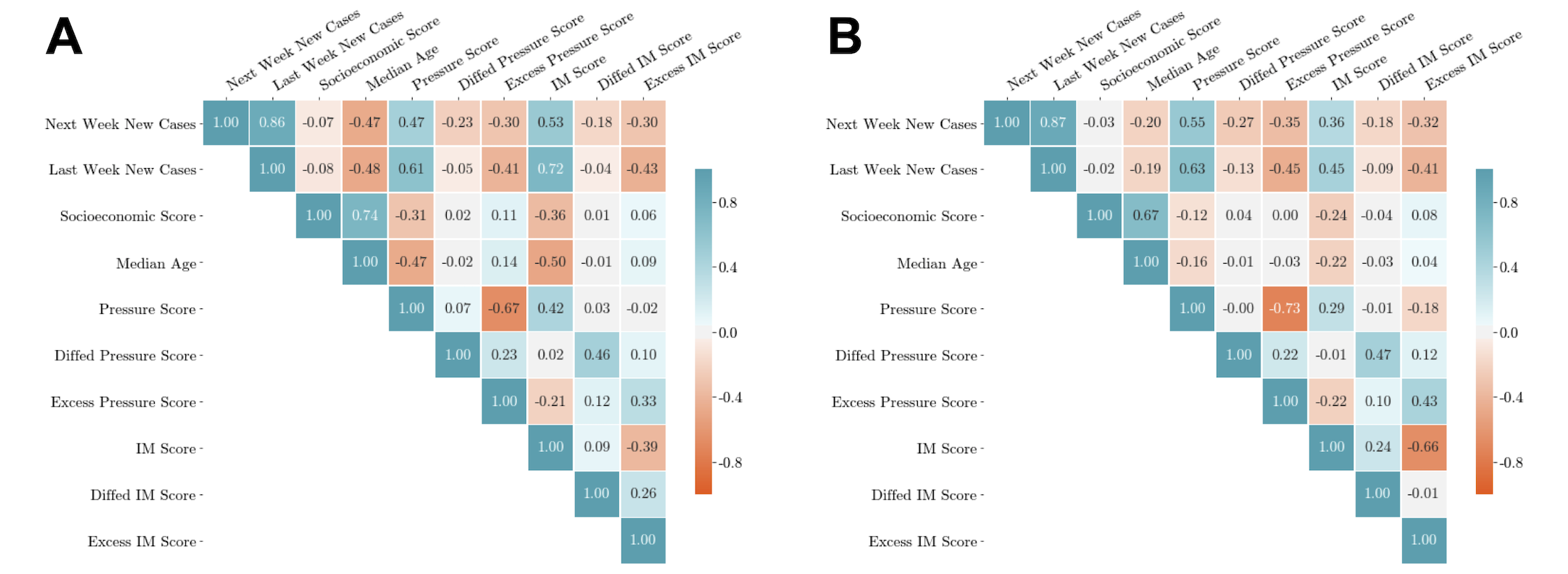

Supplement: S2 Fig — New cases and the Pressure and Internal Movement Scores are per 100,000 people. (TIF) [file pone.0253865.s002.tif]

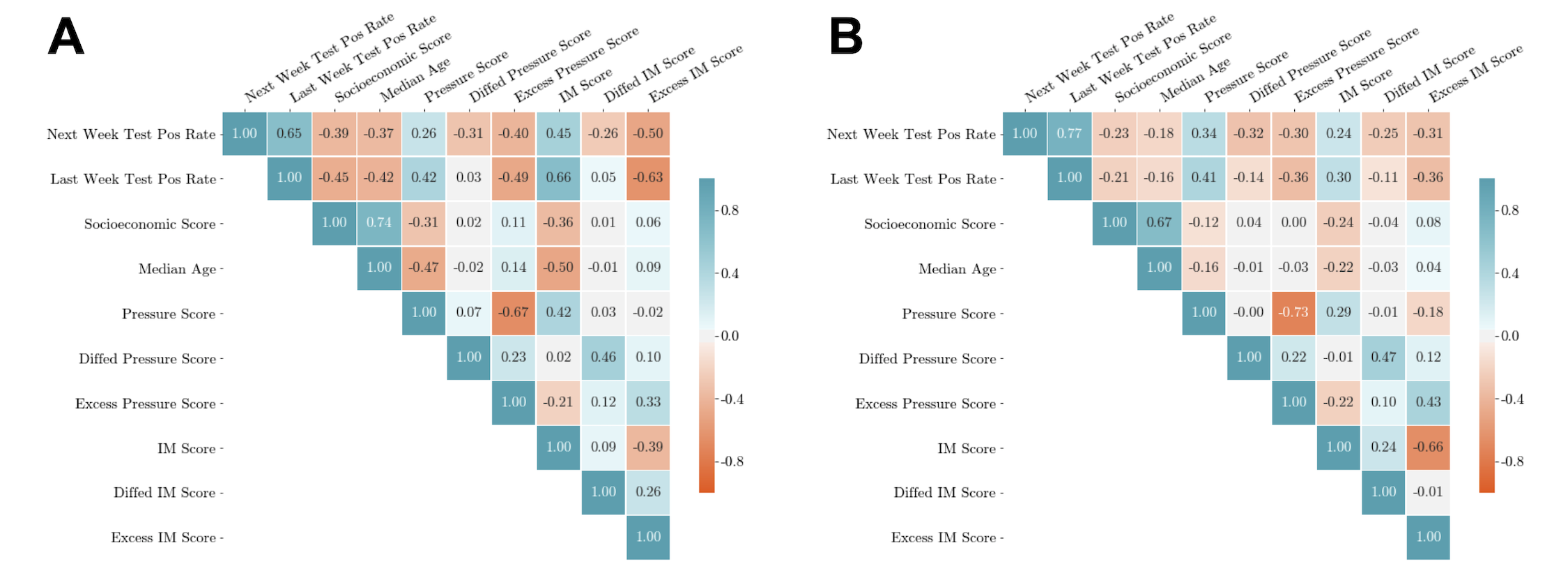

Supplement: S3 Fig — The Pressure and Internal Movement Scores are per 100,000 people. (TIF) [file pone.0253865.s003.tif]

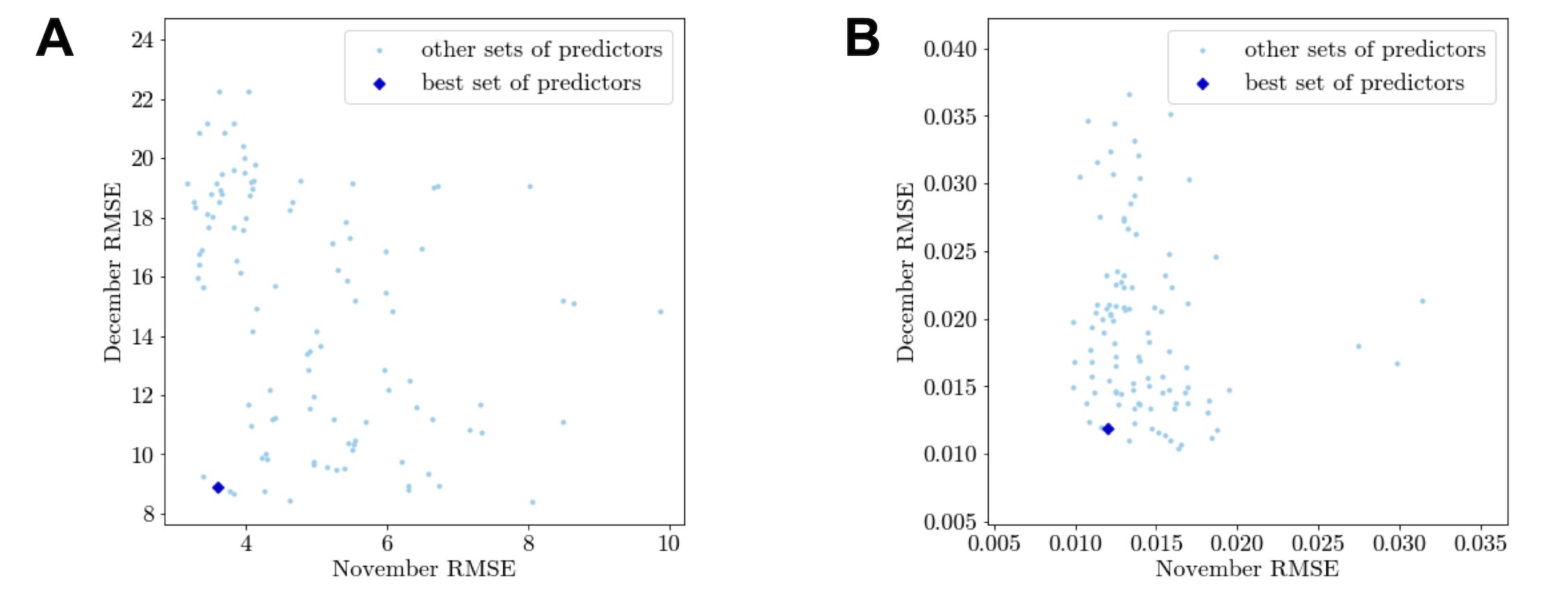

Supplement: S4 Fig — The set of predictors that minimized average RMSE over each month is shown as a dark blue diamond and is near the bottom left corner of each plot. (TIF) [file pone.0253865.s004.tif]

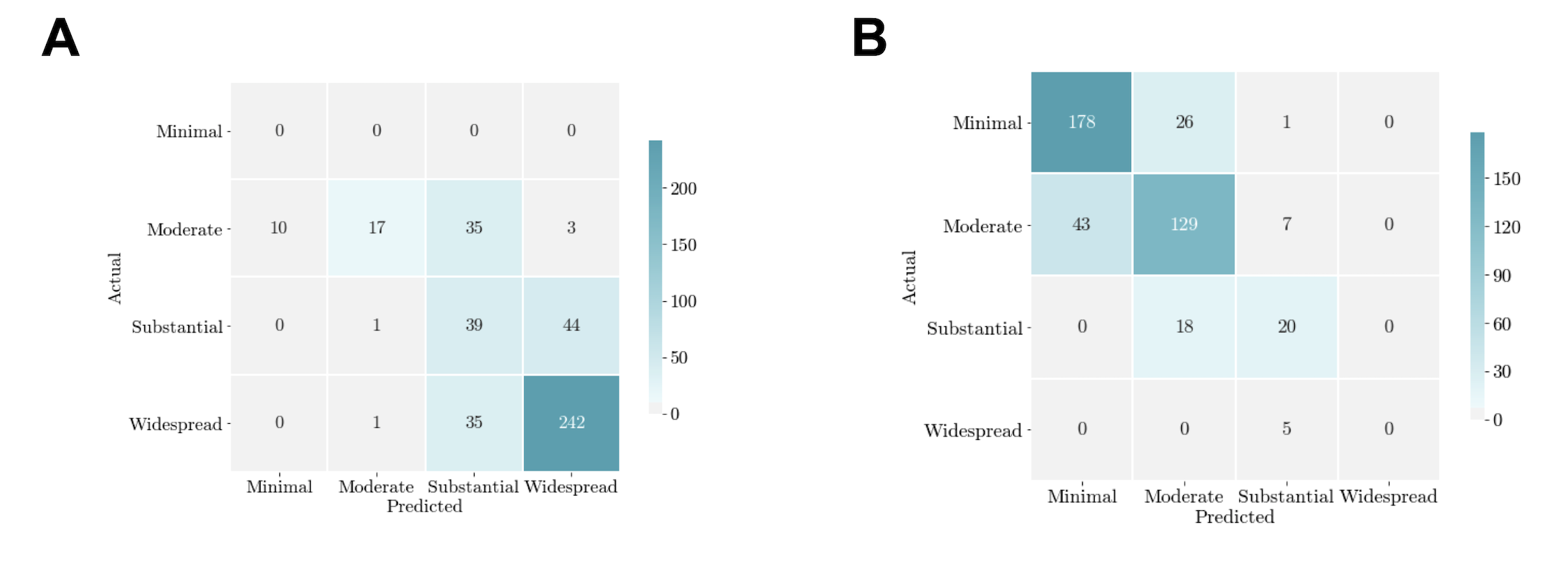

Supplement: S5 Fig — Tiers are defined by (a) new cases and (b) test positivity rate that are positive from November 1 to December 31, 2020. (TIF) [file pone.0253865.s005.tif]

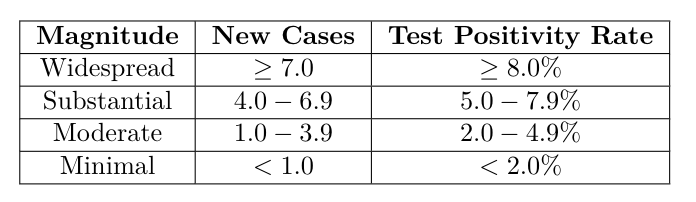

Supplement: S1 Table — (PNG) [file pone.0253865.s009.png]

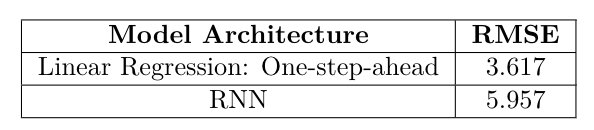

Supplement: S2 Table — (PNG) [file pone.0253865.s010.png]
