# Supplementary material for: Early detection of COVID-19 outbreaks using human mobility data
Source: PLoS One. 2021 Jul 20;16(7):e0253865. doi: 10.1371/journal.pone.0253865 (PMC8291683; doi:10.1371/journal.pone.0253865)
Supplement: S1 Appendix — (PDF) [file pone.0253865.s006.pdf]

S1 Appendix. Synthetic Mobility Data for December 2020. Because the mobility dataset ended on November 30, 2020, we generated synthetic mobility data for December 1-31, 2020. For each date in December, we identified dates that had similar mobility restrictions earlier in the pandemic (“similar dates”). We validated the similarity of restrictions between dates by a routine index presented by one of the leading news companies in Israel [39]. The index uses the number of commuters on public transportation, the unemployment rate, and the rate of expenses, among other factors, to represent the similarity between mobility on any given day and a pre-COVID-19 “routine” day, where a score of 100% reflects routine mobility before the pandemic. The first week of December was similar to the last week of November, the middle three weeks of December were similar to July, and the last five days of December were similar to the third and fourth weeks of September. We matched each date in December to the corresponding similar date(s) by the day of the week. Then, we estimated mobility data for the December dates by perturbing averaged mobility data over their similar dates. We either added or subtracted a random noise element centered around 0 with a standard deviation of 25% of the similar dates’ values, based on whether the restrictions were exactly the same (positive or negative perturbation) or less severe (positive perturbation; more mobility). For example, if all restrictions were the same on days A and B, except that schools were open on day B, we would apply a positive perturbation to day A’s mobility data to estimate mobility on day B.

References

39. Return to routine index; 2021. Available from: `https://corona.mako.co.il/?item=back-to-normal-graph-canvas`.
