# Supplementary material for: Early detection of COVID-19 outbreaks using human mobility data
Source: PLoS One. 2021 Jul 20;16(7):e0253865. doi: 10.1371/journal.pone.0253865 (PMC8291683; doi:10.1371/journal.pone.0253865)
Supplement: S2 Appendix — (PDF) [file pone.0253865.s007.pdf]

S2 Appendix. Correlations between Model Inputs and Outputs. Lagged health features were most strongly correlated with the same health features in the next 7 days. These correlations are independent of the mobility data. A past week's 7-day average of new cases was strongly correlated with the following week's 7-day average of new cases ($r = 0.86$ and $r = 0.87$ during the actual and extended mobility periods, respectively; S2 Fig), and a past week's test positivity rate was strongly correlated with the next week's average test positivity rate ($r = 0.65$ and $r = 0.77$ during the actual and extended mobility periods, respectively; S3 Fig). Because of the strong correlations between lagged health features and our intended predictors, we included 1-day, 3-day, and 6-day lagged health features as potential predictors for each prediction task.

We found lagged correlation between inter-district travel and COVID-19 incidence. In particular, if district A has a high number of COVID-19 cases and there has been significant travel from district A to district B over the past week, then we find that district B will have a relatively high number of new COVID-19 cases in the following week. The Pressure Score for a district from the past 7 days was correlated with new cases in the district averaged over the next 7 days, with $r = 0.47$ (actual mobility period) and $r = 0.66$ (extended mobility period). The Internal Movement Score for a district from the past 7 days was also correlated with new cases averaged over the next week, with $r = 0.53$ (actual mobility period) and $r = 0.36$ (extended mobility period). Similarly, the average test positivity rate over the next week was correlated with the Pressure Score from the past 7 days, with $r = 0.26$ (actual mobility period) and $r = 0.34$ (extended mobility period), and also correlated with the Internal Movement Score from the past 7 days with $r = 0.45$ (actual mobility period) and $r = 0.24$ (extended mobility period). We therefore included the Pressure and Internal Movement Scores, alone and combined, as potential predictors for all prediction tasks.

Negative correlations between a week's new COVID-19 cases and the previous week's differenced and Excess Pressure and Internal Movement Scores reflect the effects of lockdowns and other policy measures implemented to reduce COVID-19 spread. A greater reduction in mobility compared to the February 2020 baseline, as reflected by the Excess Internal Movement and Excess Pressure Scores, was correlated with a reduction in new cases over the next 7 days ($r = -0.30$ for both Excess Pressure and Internal Movement, over the actual mobility period, and $r = -0.35$ and -0.32, respectively, over the extended mobility period). The Excess Internal Movement and Excess Pressure Scores were also correlated with a reduction in test positivity rate over the next 7 days over both evaluation periods (r scores of -0.40, -0.50, -0.30, -0.31, respectively). Differenced Pressure and Internal Movement Scores produced similar results of slightly smaller magnitude. As we did for the Pressure and Internal Movement Scores, we considered the excess and differenced mobility predictors separately and together as predictors for all prediction tasks.

Because we predicted cases within each district separately, we did not include socioeconomic score or median age, which are constant factors, as potential features when predicting new cases and the test positivity rate. However, we note that socioeconomic score was negatively correlated with the test positivity rate: poorer districts tended to have higher test positivity rates ($r = -0.39$ and $r = -0.23$ during the actual and extended mobility periods, respectively). Median age was correlated with new cases ($r = -0.47$ and $r = -0.20$ during the two periods, respectively) but socioeconomic score was not correlated with new cases ($r = -0.07$ and $r = -0.03$ during the respective periods).
