# Supplementary material for: Early detection of COVID-19 outbreaks using human mobility data
Source: PLoS One. 2021 Jul 20;16(7):e0253865. doi: 10.1371/journal.pone.0253865 (PMC8291683; doi:10.1371/journal.pone.0253865)
Supplement: S3 Appendix — (PDF) [file pone.0253865.s008.pdf]

S3 Appendix. Predictions: Other Model Types. RNNs capture temporal behavior in data and can use past steps as input to generate output sequences [40]. One RNN architecture that has had success in clinical time series applications, such as accurately forecasting outpatient clinic demand [41, 42], is the long short-term memory (LSTM) architecture. Past literature has explored using RNNs and LSTMs for predicting accumulated cases of COVID-19 [13–17]. Our network structure composed an RNN with one hidden LSTM layer of 8 units; this structure performed the best on the validation districts. We trained our LSTM for 1,000 epochs with an early stopping function to pause training if validation mean squared error had not decreased for 5 epochs. When training the LSTM, we took out a validation period from our training interval from September 16 – October 24, 2020. We implemented this model with TensorFlow [43].

We evaluated these models' predictions of new cases during the period for which we had actual mobility data, November. One-step-ahead linear regression models outperformed the RNN (S2 Table), most likely because RNNs require larger amounts of data to be trained optimally.

References

13. Kırbaş I, Sözen A, Tuncer AD, Kazancıoğlu FS. Comparative analysis and forecasting of COVID-19 cases in various European countries with ARIMA, NARNN and LSTM approaches. Chaos Solitons Fractals. 2020;138:110015.

14. Bodapati S, Bandarupally H, Trupthi M. COVID-19 time series forecasting of daily cases, deaths caused and recovered cases using long short term memory networks. In: 2020 IEEE 5th International Conference on Computing Communication and Automation (ICCCA); 2020. p. 525–530.

15. Shahid F, Zameer A, Muneeb M. Predictions for COVID-19 with deep learning models of LSTM, GRU and Bi-LSTM. Chaos Solitons Fractals. 2020;140:110212.

16. Shastri S, Singh K, Kumar S, Kour P, Mansotra V. Time series forecasting of Covid-19 using deep learning models: India-USA comparative case study. Chaos Solitons Fractals. 2020;140:110227.

17. Arora P, Kumar H, Panigrahi BK. Prediction and analysis of COVID-19 positive cases using deep learning models: A descriptive case study of India. Chaos Solitons Fractals. 2020;139:110017.

40. Graves A. Generating sequences with recurrent neural networks; 2013. Available from: `https://arxiv.org/abs/1308.0850`.

41. Guan G, Engelhardt BE. Predicting sick patient volume in a pediatric outpatient setting using time series analysis. In: Proceedings of Machine Learning Research. vol. 106; 2020. p. 271–287.

42. Lipton ZC, Kale DC, Elkan C, Wetzel RC. Learning to diagnose with LSTM recurrent neural networks. In: Proceedings of 4th International Conference on Learning Representations; 2016. p. 1–18.

43. TensorFlow: An end-to-end open source machine learning platform; 2021. Available from: `https://www.tensorflow.org/`.
